# Selective detection of Mg^2+^ ions via enhanced fluorescence emission using Au–DNA nanocomposites

**DOI:** 10.3762/bjnano.8.79

**Published:** 2017-04-03

**Authors:** Tanushree Basu, Khyati Rana, Niranjan Das, Bonamali Pal

**Affiliations:** 1 School of Chemistry and Biochemistry, Thapar University, Patiala 147004, Punjab, India; 2 Department of Biotechnology, Thapar University, Patiala 147004, Punjab, India

**Keywords:** Au–DNA nanocomposites, enhanced fluorescence emission, metal-ion detection, Mg^2+^ ion detection

## Abstract

The biophysical properties of DNA-modified Au nanoparticles (AuNPs) have attracted a great deal of research interest for various applications in biosensing. AuNPs have strong binding capability to the phosphate and sugar groups in DNA, rendering unique physicochemical properties for detection of metal ions. The formation of Au–DNA nanocomposites is evident from the observed changes in the optical absorption, plasmon band, zeta potential, DLS particle size distribution, as well as TEM and AFM surface morphology analysis. Circular dichroism studies also revealed that DNA-functionalized AuNP binding caused a conformational change in the DNA structure. Due to the size and shape dependent plasmonic interactions of AuNPs (33–78 nm) with DNA, the resultant Au–DNA nanocomposites (NCs) exhibit superior fluorescence emission due to chemical binding with Ca^2+^, Fe^2+^ and Mg^2+^ ions. A significant increase in fluorescence emission (λ_ex_ = 260 nm) of Au–DNA NCs was observed after selectively binding with Mg^2+^ ions (20–800 ppm) in an aqueous solution where a minimum of 100 ppm Mg^2+^ ions was detected based on the linearity of concentration versus fluorescence intensity curve (λ_em_ = 400 nm). The effectiveness of Au–DNA nanocomposites was further verified by comparing the known concentration (50–120 ppm) of Mg^2+^ ions in synthetic tap water and a real life sample of Gelusil (300–360 ppm Mg^2+^), a widely used antacid medicine. Therefore, this method could be a sensitive tool for the estimation of water hardness after careful preparation of a suitably designed Au–DNA nanostructure.

## Introduction

The interactions between Au nanoparticles (AuNPs) and DNA are essential to classify and expand upon, given the potential applications for NP–DNA complexes such as gene therapy, drug delivery, and DNA decoding. The importance of AuNPs is due to their unique optical properties related to the collective oscillation of the surface electrons, called surface plasmonic resonance (SPR) [1]. Since the frequency of this SPR band depends on the size, shape and chemical environment of the AuNP, any change in the environment of these particles, such as adsorption, desorption or aggregation, will shift the SPR band frequency. Given the collective oscillation, Au nanostructures can act as signal intensifiers and lead to enhancement of the fluorescence and scattering response in various DNA detection schemes. The high sensitivity of the plasmon spectra towards the particle size and the local dielectric environment also offers new methods for the detection of free DNA or other biomolecules [2], where the detection signal is exclusively based on the color changes during assay or modifications in the plasmonic spectra.

The biophysical properties of DNA make it compatible for linkage with metals, which are useful in a variety of applications such as biosensor development. They can also be stabilized with a wide variety of molecules because of the alkyl thiol adsorption phenomena [3]. Also, in DNA, the specific base pairing and the availability of free hydroxyl and phosphate groups have been used to build the structured assembly of particles [4]. DNA-functionalized Au nanoparticles are often applied as nanoscale building blocks in assembly strategies, nanotherapeutics and antisense agents [5].

Currently, most of the research activities on Au–DNA nanocomposites (NCs) are in the field of therapeutics, medicine, and gene therapy. For example, in 2014, Li and co-workers reported the detection of the heavy metals Hg^2+^ and Cu^2+^ using a DNA–Ag nanocomposite system [6]. They demonstrated the change in fluorescence quenching due to the interaction of the DNA–Ag nanocluster with Hg^2+^ ions in water. Also, Ma and co-workers reported the emission modulation of DNA-templated fluorescent Ag nanocomposites by divalent Mg^2+^ ions in 2011 [7]. Presently, there are many biosensors which are based on different sensing mechanisms [8]. The influence of the size and shape of the AuNPs on their optical properties had been well studied, but there has been less data generated on the alteration of the DNA conformation due to changes in the size and shape of the nanoparticles. Moreover, the effect on the optical activity of AuNPs in the presence of double-stranded DNA has not been extensively explored for its practical applications.

**Scheme 1:**
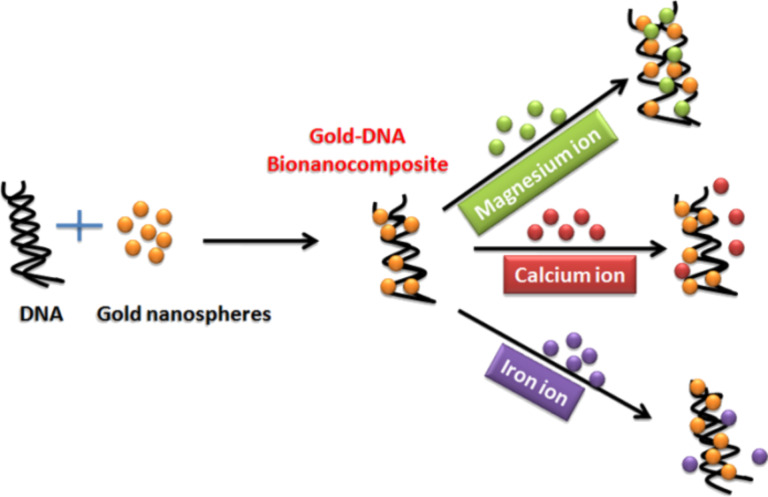
Au–DNA nanocomposite interactions with Mg^2+^, Ca^2+^ and Fe^2+^ ions.

Alkali and transition metals have been significantly studied with regard to their physical activity on biological systems as well as environmental processes [9]. Mg^2+^ is an essential mineral nutrient present in the environment, every cellular organism, and in many kinds of medicines. However, an excess amount of such metal is poisonous and can result in a series of health or environmental problems. Magnesium is the fourth most abundant metal ion present in nature. However, at higher concentrations in the body, it causes severe damage to the gastrointestinal tract (GIT), liver and heart. Some methods employing radioactive isotopes [10–11], fluorescent indicators [12–14] and electrophysiology [12,15] have been used for the detection of Mg^2+^ ions in biological samples. These, however, are time consuming and expensive. However, very little information is available regarding the optical properties and fluorescence intensity of Au–DNA nanocomposites (NCs) utilized for metal-ion sensing. The present study demonstrates (Scheme 1) how the preparation and characterization of Au–DNA NCs can be used to tune their optical properties and fluorescence emission for the detection of Mg^2+^, Ca^2+^ and Fe^2+^ ions.

As there is a difference in the ionic size and linkage of metal ions with Au–DNA NCs, the fluorescence properties can be significantly varied depending on the nature of the metal ion interaction [16]. The Au–DNA NC was found to be fairly effective for the detection of Mg^2+^ ions as compared to Ca^2+^ and Fe^2+^ ions present in aqueous solution. The enhanced binding ability is likely due to the smaller size of the Mg^2+^ ions.

## Results and Discussion

**Figure 1:**
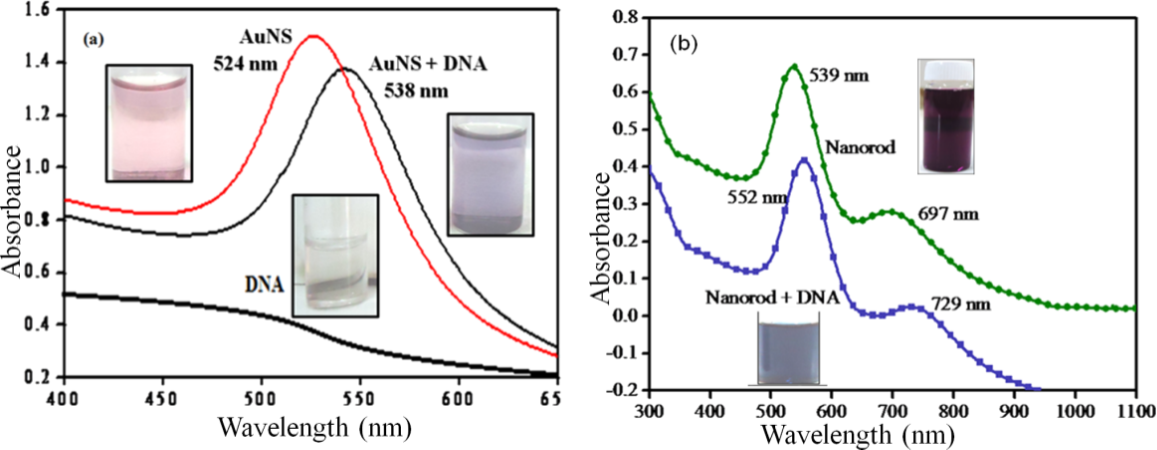
Surface plasmon absorption band of (a) Au nanospheres and (b) Au nanorods before and after DNA modification.

The optical absorption of different bare and DNA-modified AuNPs (AuNS-1, AuNS-2, AuNS-3 and AuNS-4) is shown in Supporting Information File 1, Figure S1. It was found that due to their size dependency [17], the plasmon band shift (summarized in Supporting Information File 1, Table S1) occurred towards longer wavelengths (λ_max_ varied from 524 to 637 nm) [18–21]. The sample AuNS-4 exhibited a maximum shift (λ_max_ ≈ 15 nm) relative to other nanospheres as these larger sized particles exhibit more light scattering. The AuNS-1 sample resulted in a shift from 524 to 538 nm upon addition of DNA, while DNA does not show any band in this region as seen in Figure 1a.

**Figure 2:**
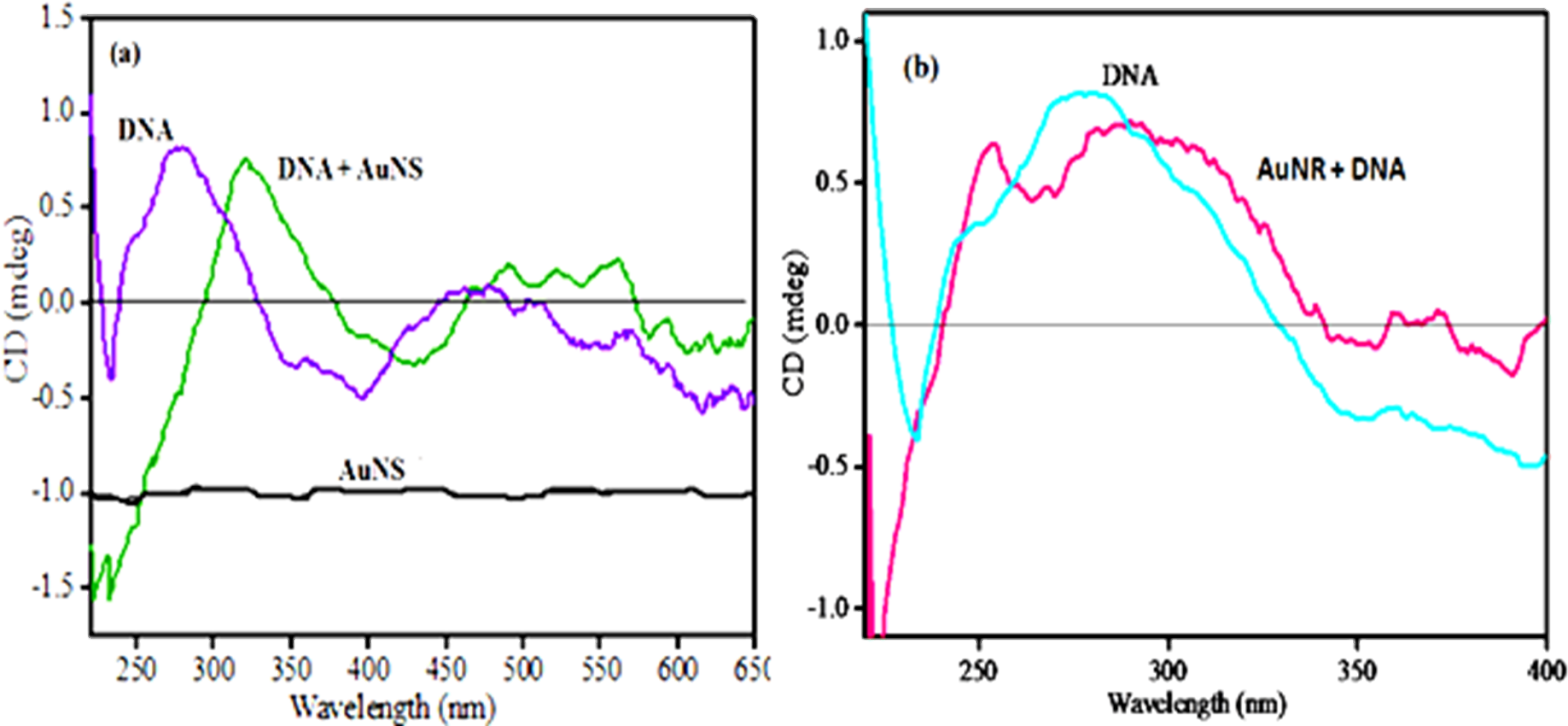
Circular dichroism spectral changes in the DNA conformation upon binding with (a) Au nanospheres and (b) Au nanorods.

The anisotropic Au nanorods (AuNRs) also displayed a red shift in the transverse and longitudinal bands after DNA addition, as shown in Figure 1b [22–24]. The shift in λ_max_ was 13 nm and 28 nm for transversal and longitudinal peaks, respectively. The loading of DNA could be maximized along the longitudinal cross-section of the AuNR, therefore the major red shift was observed in this region, probably due to the close contact of dispersed AuNPs with the addition of DNA [25]. The super asymmetry of the DNA helix gives rise to degenerate interactions between chromophoric bases, resulting in intense circular dichroism (CD) spectra. The DNA in its characteristic right-handed B form exhibits an absorbance spectrum in the far UV region (220–320 nm). The free DNA showed a negative peak at 247 nm and a positive peak at approximately 278 nm in the CD spectrum, which corresponds to B-DNA. These observations were due to stacking interactions between the bases and the helical structure of DNA [26]. As shown in Figure 2a, upon addition of Au nanospheres (AuNS) to the DNA solution, the molar ellipticity decreased at approximately 220 nm and increased by approximately 280 nm [27]. These changes, coupled with a shift in the maximum wavelength of the positive band, indicated partial denaturation [28]. The same changes were observed when AuNRs were added to a DNA solution as shown in Figure 2b. This indicated the conformational changes in DNA upon binding with AuNPs.

**Figure 3:**
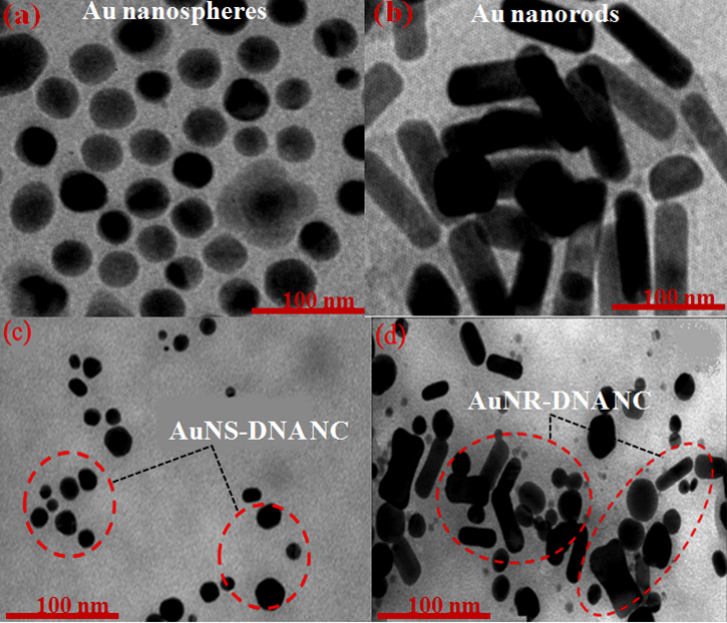
TEM images of bare (a) AuNSs, (b) AuNRs, (c) AuNS–DNA and (d) AuNR–DNA nanocomposites.

The TEM images of AuNSs and AuNRs are shown in Figure 3, which reveal the formation of Au–DNA NCs [29]. These AuNSs were found to be separated from each other due to the CTAB coating on their surface, which renders them to have a positive charge [30].

**Figure 4:**
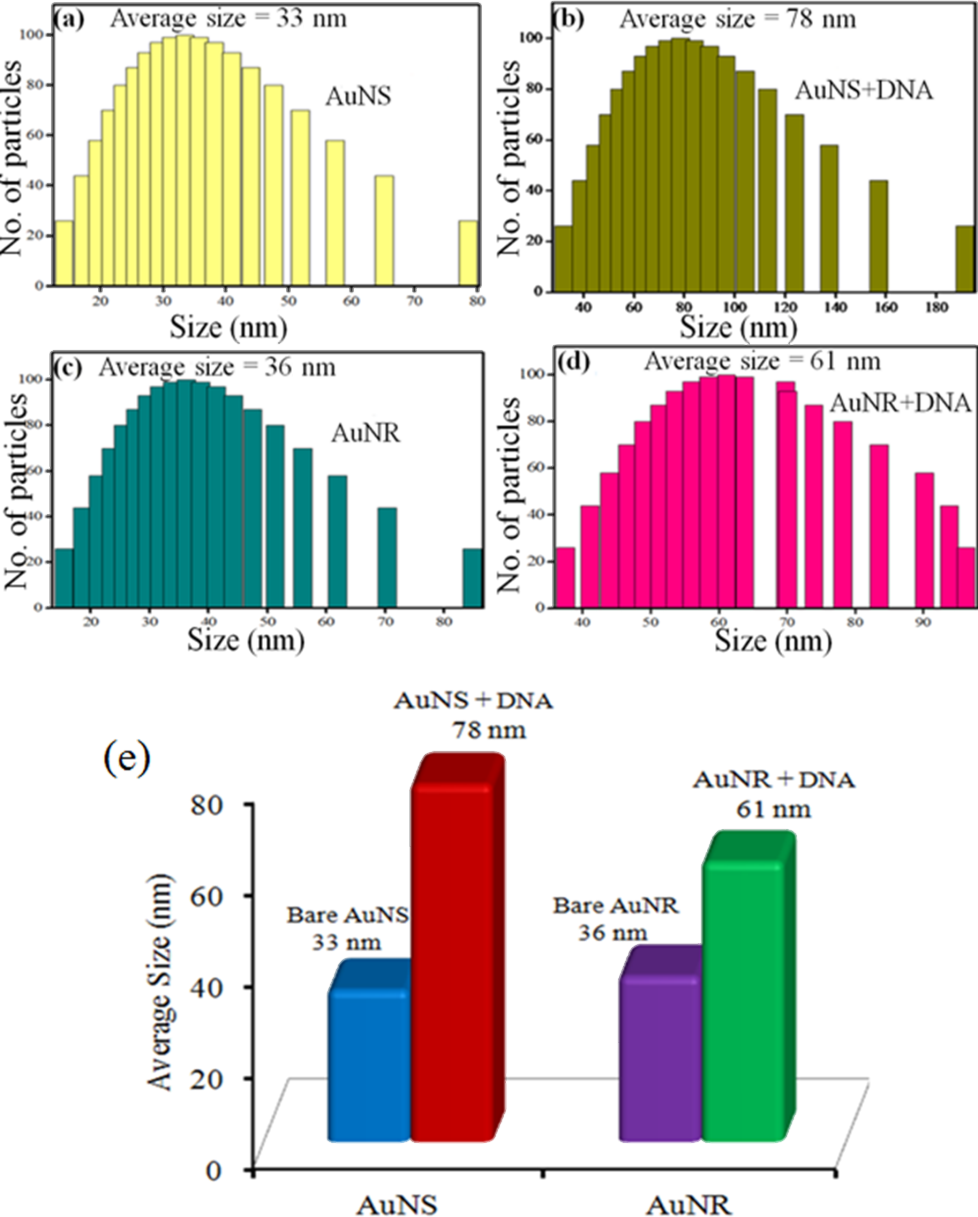
Dynamic light scattering particle size distribution of bare and DNA modified (a,b) AuNS-1, (c,d) AuNRs and (e) their comparative average diameter data.

The dynamic light scattering (DLS) results illustrated in Figure 4 show that after the binding of DNA to AuNPs, the diameter of the structures in AuNS-1 increased from 33 to 78 nm and for AuNR it increased from 36 to 61 nm. It was concluded that upon binding of AuNPs with DNA, a particular pattern was followed and there was a complex formation of DNA with AuNS and AuNR [31]. The complex formed was considered to be a Au–DNA NC. This increase in diameter confirmed the binding of DNA to different AuNSs and AuNRs.

**Figure 5:**
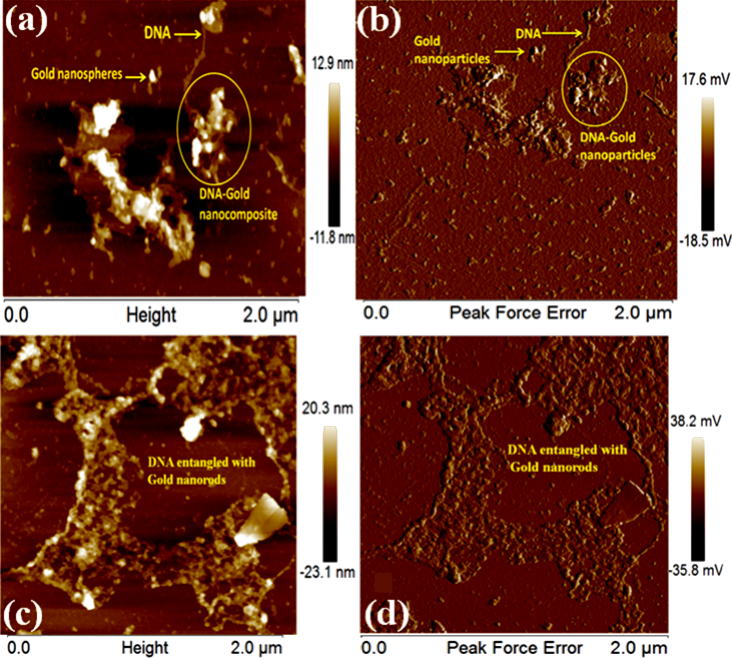
AFM images of (a,b) AuNS 1-DNA nanocomposites and (c,d) AuNR–DNA nanocomposites showing height and amplitude mode.

The AFM images also confirmed the binding of functionalized DNA with AuNPs. Although the images alone were not enough evidence, together with the data obtained from literature, they show the change in position of the AuNSs and AuNRs [32]. Figure 5 clearly shows the even distribution of gold nanoparticles in the solution. The AFM images in Figure 5a show the distribution of AuNSs on the silicon wafer which indicates the proper dispersion of AuNSs in the solution. The height range was taken from 0–2 μm. The images as shown in Figure 5a confirmed the presence of DNA. In the solution, DNA was clearly identified as a thread-like structure. The complex of AuNS–DNA was also observed which led to the structural confirmation of binding of functionalized DNA with AuNPs. Figure 5b shows that the amplitude range obtained for Au–DNA NCs was higher than the amplitude range for only AuNSs, which is summarized in Supporting Information File 1, Figure S2b.

The appearance of entangled DNA is shown in Figure 5c. This morphology might be due to the clustering of gold nanorods around the DNA. As shown in Figure 5, the height of the sample from the surface was estimated to be around 20.3 nm. The brighter areas on the image had a maximum height of 20.3 nm. Figure 5d shows the amplitude of DNA–AuNR to be 38.2 mV.

**Figure 6:**
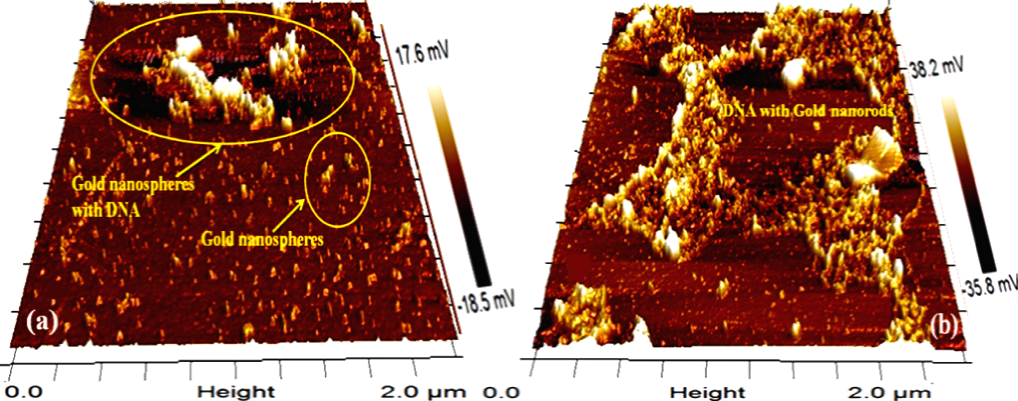
3D AFM images of Au–DNA nanocomposites of (a) AuNSs and (b) AuNRs.

Figure 6 shows the 3D visualization of the structures. AuNSs are well-dispersed as shown in Figure 6a, but clusters appeared on the upper part on the slide which were considered to be Au–DNA nanocomposites. Likewise, the clustering of DNA and AuNRs is shown in Figure 6b.

**Figure 7:**
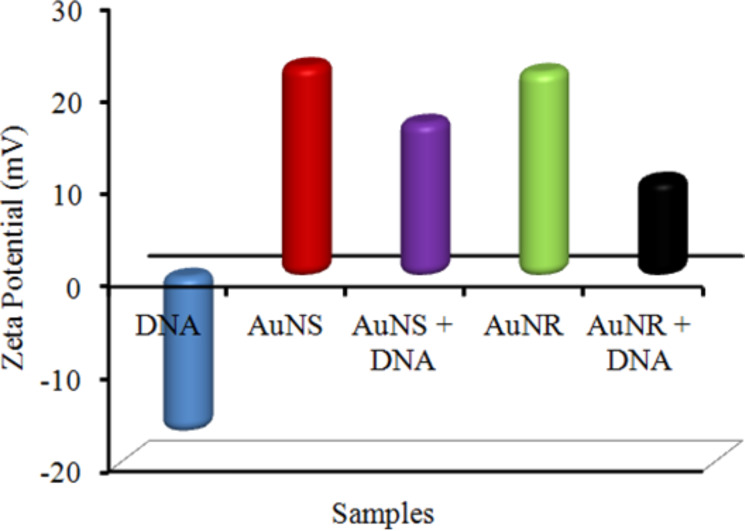
Variation in zeta potential of AuNPs before and after DNA modification.

The negative charge of DNA slightly decreased upon binding with mercapto propionic acid (MPA). The synthesized AuNSs had a CTAB coating and were positively charged in nature. The values obtained for the zeta potential, conductance and mobility are summarized in Supporting Information File 1, Table S2. In Figure 7, the zeta potential is given before binding with DNA for bare AuNSs and AuNRs to be +22.8 mV and +26.16 mV, respectively. However, zeta potential of DNA was −16.84 mV and upon binding with AuNSs and AuNRs changed to +16.67 mV and +10.40 mV, respectively.

It was observed that after DNA–AuNP binding, the resultant electronic charge of the Au–DNA nanocomposite is neutralized to some extent, as previously reported [33]. In addition, the electrostatic repulsion between positive surface charges of Au–DNA composites also imparts stability to a considerable extent. Along with changes in zeta potential, changes in mobility and conductance were also observed. This gives an idea about the electrostatic interactions between DNA and AuNPs.

### Metal-ion detection

**Figure 8:**
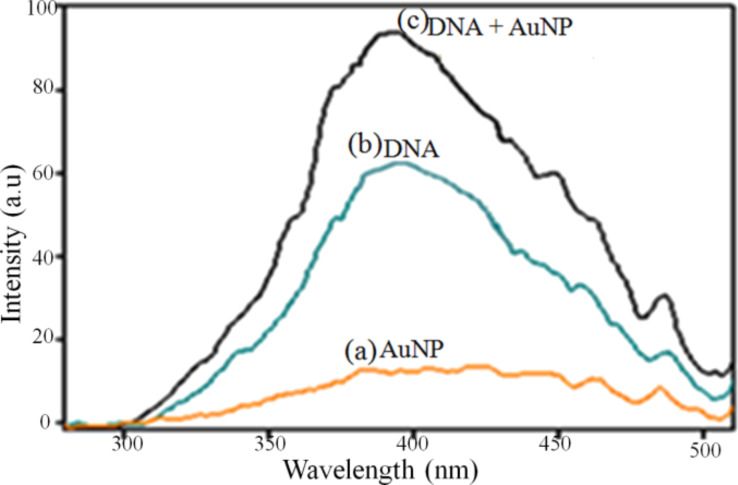
Fluorescence emission intensity of bare (a) AuNPs, (b) DNA and (c) AuNP–DNA composites.

Upon excitation at 260 nm, the AuNPs did not exhibit fluorescence, however, bare herring sperm DNA used for detection showed fluorescence emission at 400 nm. A significant increase in the fluorescence emission (λ_ex_ = 260 nm) of Au–DNA nanocomposites was observed due to the charge transfer between AuNP–DNA interfaces as seen in Figure 8. Also, the surface plasmonic absorption of AuNPs resulted in an increased charge transfer between the AuNPs and the DNA surface [34].

The Au–DNA NC material was used as a system to detect the presence of metal ions in a solution. Calcium (Ca), iron (Fe) and magnesium (Mg) were chosen because of their high abundance and importance in nature.

**Figure 9:**
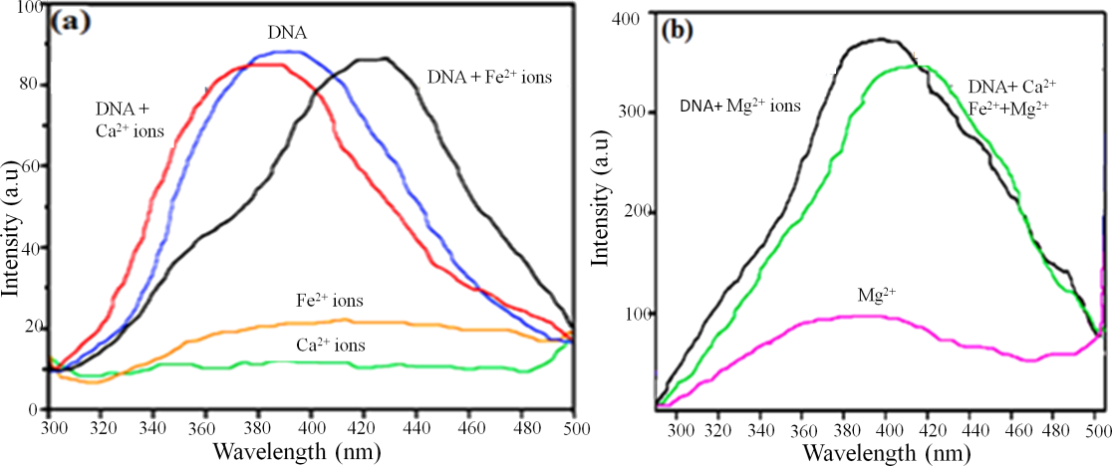
Change in fluorescence intensity for a sample with (a) Fe^2+^ and Ca^2+^ ions and (b) Mg^2+^ ions, with and without DNA binding.

Au–DNA NCs are a convenient and time-effective approach to metal detection. As can be observed in Figure 9a, the detection of Ca^2+^ did not produce any fluorescence, and even upon adding Ca^2+^ to the herring sperm DNA solution, there was no change in the fluorescence of DNA. The intensity of bare DNA was comparable with the sample where calcium ions were treated with DNA. Similarly to the calcium ions, Fe^2+^ ions did not significantly affect the fluorescence intensity.

As shown in Figure 9b, there was an increase in the intensity of DNA upon addition of Mg^2+^ ions in the DNA solution. Attempts were made to determine whether DNA binding could be used to detect Mg^2+^ ions in the presence of other two metal ions. When all three metal ions and DNA were combined, it was observed that the fluorescence intensity was similar to that of only Mg^2+^ ions. This indicated that the system could selectively detect Mg^2+^ ions.

**Figure 10:**
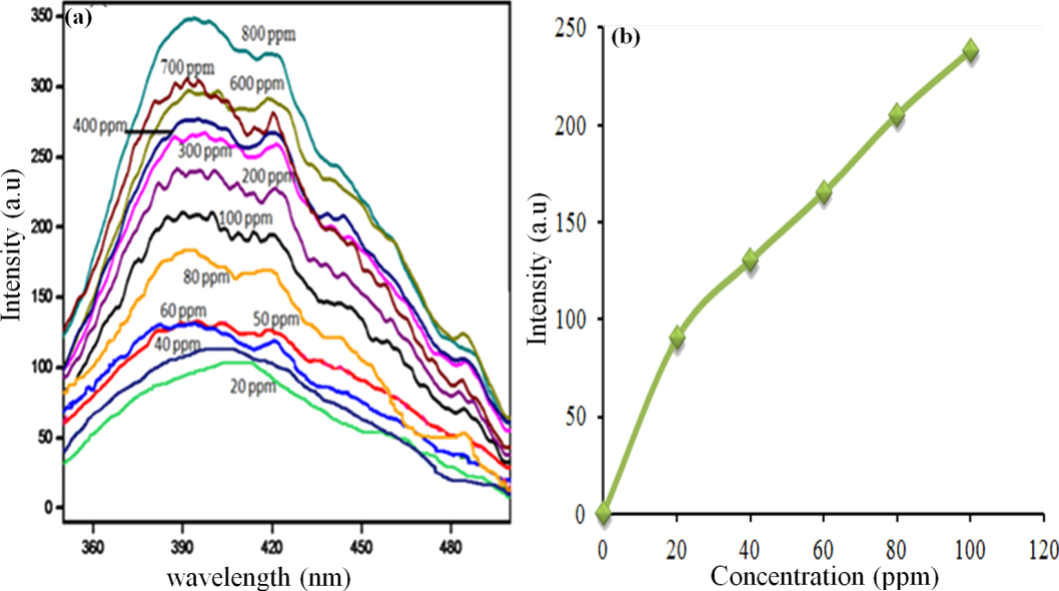
(a) Fluorescence intensity of Au–DNA nanocomposites with increasing concentration (ppm) of Mg^2+^ and (b) the calibration curve of the fluorescence intensity vs concentration (ppm) of Mg^2+^ ions.

The prepared Au–DNA nanocomposites under study were mixed with varying concentrations of Mg^2+^ ions for evaluation of the interaction between Au–DNA NCs and Mg^2+^ by fluorescence emission spectra, as summarized in Supporting Information File 1, Figure S3. The fluorescence intensity gradually increases with increasing Mg^2+^ ion concentration. The fluorescence intensity of the Au–DNA NCs was measured using a wide range of Mg^2+^ ion concentrations (20 to 800 ppm as shown in Figure 10) which revealed its lowest detection limit.

### Analysis of real life samples

**Figure 11:**
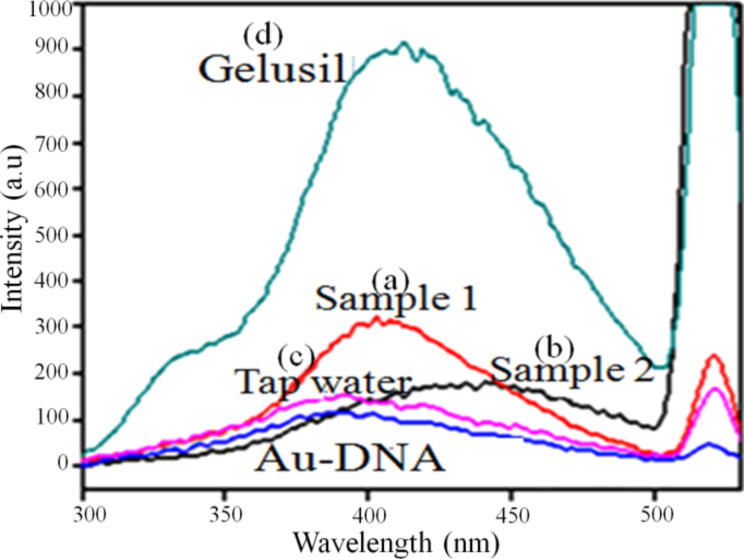
Fluorescence emission of an aqueous solution of Mg^2+^ ions in Sample 1 (50 ppm), Sample 2 (150 ppm), tap water and an aqueous solution of gelusil (300 ppm) in comparison to bare Au–DNA nanocomposite.

To analyze the activity of the nanocomposite system, the detection of Mg^2+^ in different real life samples was performed. Laboratory tap water and Gelusil (a commercial antacid) were taken for analysis. The sample preparation and procedure are described in the section Metal-ion detection. Some samples with unknown concentration were also used to check the viability of this sensing process. It was observed from the calibration curve (summarized in Supporting Information File 1, Figure S4) that the Au–DNA NC material used as sensor for Mg^2+^ ion detection was effective only upto 100 ppm. From Figure 11 it was observed that the fluorescence intensity of Gelusil was 900 a.u. From the calibration curve as shown in Figure 10b the fluorescence intensity was found to be 238 a.u. for 100 ppm concentration of Mg^2+^ ions. Hence, by using this relationship between intensity and concentration, we calculated the concentration of Mg^2+^ ion in Gelusil, which was 360 ppm (the manufacturer packaging indicated 300 ppm).

This slight variation might be due to the presence of other interfering ions present in the medicine. Similarly, using the Au–DNA NC detection system, we determined the amount of Mg^2+^ present in the tap water. It was obtained as 52 ppm by converting the intensity into concentration using the values of the calibration curve. The concentration values as obtained from the calibration curve were similar to their real values, as summarized in Supporting Information File 1, Table S3.

**Figure 12:**
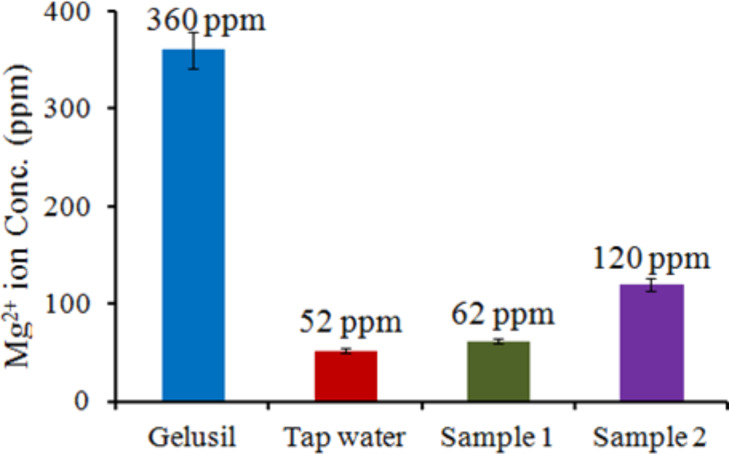
Measured concentration of Mg^2+^ ions in real life samples. The error bars represent ±5% error.

The two samples with the known Mg^2+^ ion concentration were used to cross check our detection system. In Sample 1, 60 ppm of Mg^2+^ ions was added and through fluorescence the concentration obtained was 80 ppm; for Sample 2, 150 ppm Mg^2+^ ions was added and 120 ppm was obtained through the calibration curve. All the experiments were repeated three times with ±5% error and the results are shown in Figure 12.

## Conclusion

In summary, Au–DNA nanocomposites of different shapes were synthesized and characterized through various techniques. The enhancement in the fluorescence intensity of DNA upon binding to AuNPs was taken into consideration for the nanocomposites. Using this enhanced optical property, the nanocomposite was used for metal-ion detection. It was found that the system was quite useful for the selective detection of Mg^2+^ in a mixture containing Ca^2+^, Fe^2+^ and Mg^2+^. For Ca^2+^ and Fe^2+^, no significant change in fluorescence intensity was observed; however, the intensity was increased in the presence of Mg^2+^. The selectivity towards Mg^2+^ in the mixture can be attributed to the smaller size of Mg^2+^, which aids the ionic linkage. The system is considered to be sensitive with a detection range from 20 ppm to 800 ppm for Mg^2+^ ions. The Au–DNA nanocomposite was further used to detect the presence of Mg^2+^ in tap water (50–120 ppm) and Gelusil (360 ppm), a widely used antacid medicine. Because of the simplicity, rapidity, selectivity, and reproducibility, the Au–DNA nanocomposite was successfully demonstrated as a sensing system, which holds future promise in investigating the presence of many metal ions in various environmental and industrial monitoring applications.

## Experimental

### Materials

Cetyltrimethylammonium bromide (CTAB) and DL-dithiothreitol solution (DTT) were purchased from Sigma Aldrich. Sodium borohydride (NaBH_4_) and silver nitrate (AgNO_3_) were purchased from Rankem and Fisher Scientific, respectively. Chloroauric acid (HAuCl_4_·H_2_O), ascorbic acid, mercaptopropionic acid (MPA) and magnesium acetate (Mg(CH_3_COO)_2_) were purchased from Loba Chemie. Deionized water was obtained using an ultrafiltration system (Milli-Q, Millipore) with a measured conductivity above 35 S/cm at 25 °C. DNA from herring sperm (ratio of absorbance 260 to 280 nm is 1.8) was purchased from Merck Biosciences.

### Methods

#### Synthesis of Au nanospheres and nanorods

Gold nanospheres (AuNSs) were prepared as reported previously [35]. Typically, 10 mM of HAuCl_4_·H_2_O (250 μL) and 10 mM of freshly prepared ice-cold NaBH_4_ (600 μL) solution were added to 10 mL CTAB (100 mM) with gentle mixing, and labeled as seed solution (Solution A). Further, the growth solution (Solution B) was prepared by mixing 40 mL (100 mM CTAB), 1.7 mL (10 mM HAuCl_4_·H_2_O), 250 μL of 10 mM AgNO_3_ and 270 μL (100 mM) ascorbic acid. 460 μL of Solution A was then added to Solution B to initiate the growth of nanospheres and left undisturbed for 1 h. These AuNSs served as a material which can be modified to different sizes by varying the reflux time from 2–4 h. As-prepared AuNSs were washed three times with water to remove excess CTAB, and finally dispersed in 3 mL deionized water.

Gold nanorods (AuNRs) were synthesized by the addition of 220 µL of the above prepared seed solution into the aqueous mixture (40 mL) containing CTAB (100 mM), HAuCl_4_·H_2_O (1.7 mL, 10 mM), AgNO_3_ (250 µL, 10 mM) and ascorbic acid (270 µL, 100 mM) and pH adjusted to 1–2 with 1 M HCl [36]. The synthesized AuNRs were washed three times with deionized water by centrifugation at 8500 rpm for 15 min each [37].

#### Preparation and characterization of Au–DNA nanocomposites

Herring sperm DNA (20 μL, 3.5 mg mL^−1^) was diluted with 2 mL sterile, deionized water [38] and 5 μL MPA was added to it, which was then incubated in a laminar hood at 21 °C for 24 h to ensure the binding of alkanethiol to oligonucleotides. Here, MPA helps both in the stabilization as well as thiol functionalization. MPA can easily stabilize the AuNPs due to the strong affinity of sulphur groups for gold. Also, the thiolated-DNA can directly bind to the surface of AuNPs by thiol–Au interactions [39–40]. Before use, DTT (0.1 M DTT, 10 mM phosphate buffer) was added to the above solution and again incubated at room temperature for 1 h to cleave the disulfide bonds formed by the oligonucleotides upon addition of MPA. DNA functionalized gold nanoparticles were then washed twice by centrifugation (13,000 rpm, 15 min) to remove all unbound alkanethiol double-stranded DNA from solution and re-dispersed in fresh, sterile, deionized water.

Various techniques such as UV–vis spectroscopy (using a Specord 205 spectrophotometer), circular dichroism (CD) spectroscopy (Jasco, 815), atomic force microscopy (AFM), and fluorescence spectroscopy were used to characterize AuNPs and DNA functionalized Au nanocomposites. The size and shape of the nanoparticles and nanocomposites were characterized by TEM analysis. The electrokinetic parameters such as zeta potential, hydrodynamic diameter and conductance were analyzed by a Brookhaven 7610 instrument.

## Supporting Information

File 1 Additional experimental details.
