# Supplementary material for: Selective detection of Mg2+ ions via enhanced fluorescence emission using Au–DNA nanocomposites
Source: Beilstein J Nanotechnol. 2017 Apr 3;8:762–71. doi: 10.3762/bjnano.8.79 (PMC5389202; doi:10.3762/bjnano.8.79)
Supplement: File 1 — Additional experimental details. [file Beilstein_J_Nanotechnol-08-762-s001.pdf]

Supporting Information

for

Selective detection of Mg^{2+} ions via enhanced fluorescence emission using Au–DNA nanocomposites

Tanushree Basu[1], Khyati Rana[2], Niranjan Das[2] and Bonamali Pal[1,*]

Address: [1]School of Chemistry and Biochemistry, Thapar University, Patiala 147004, Punjab, India and [2]Department of Biotechnology, Thapar University, Patiala 147004, Punjab, India

Email: Bonamali Pal - bpal@thapar.edu; Tel: +91-175-239-3491, Fax: +91-175-236-4498

* Corresponding author

Additional experimental details

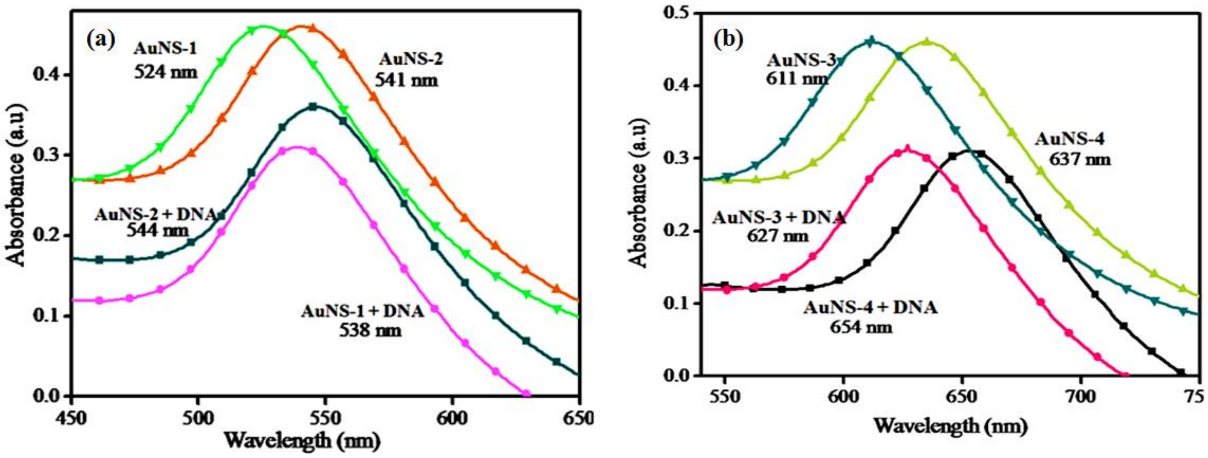

Figure S1: Shift in UV-Vis absorption spectra of different of Au nanospheres (a) AuNS-1 and AuNS-2 & (b) AuNS-3 and AuNS-4, before and after DNA addition.

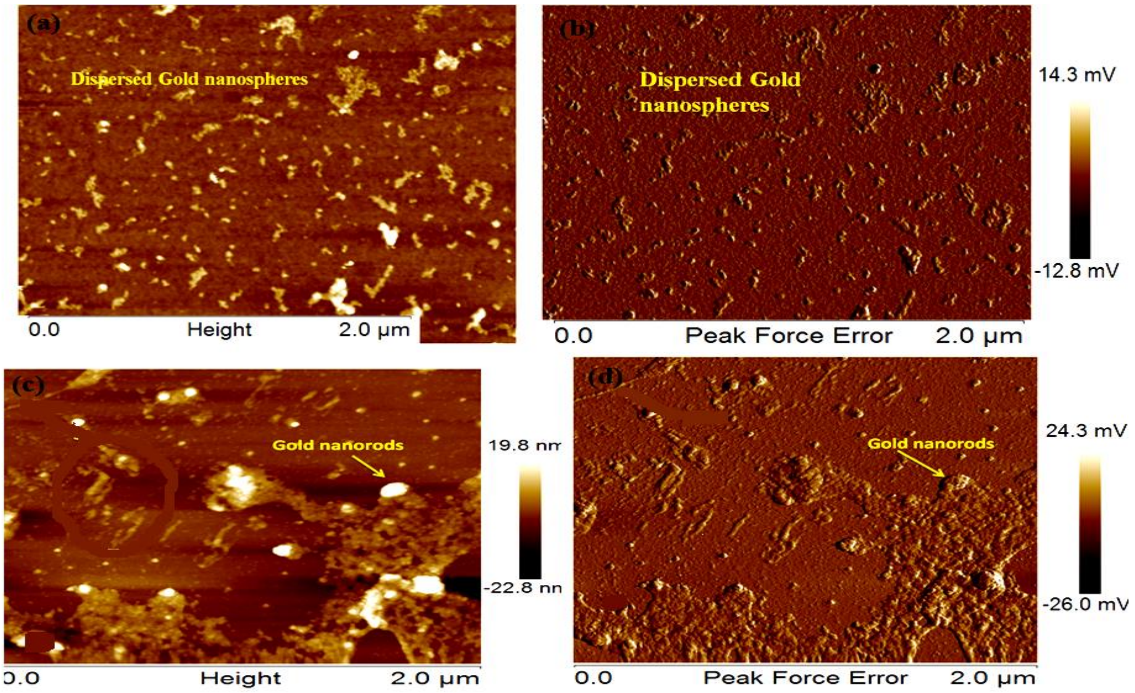

Figure S2: Images of AuNPs showing height Mode and amplitude mode (a-b) Au NS and (c-d) Au NRs.

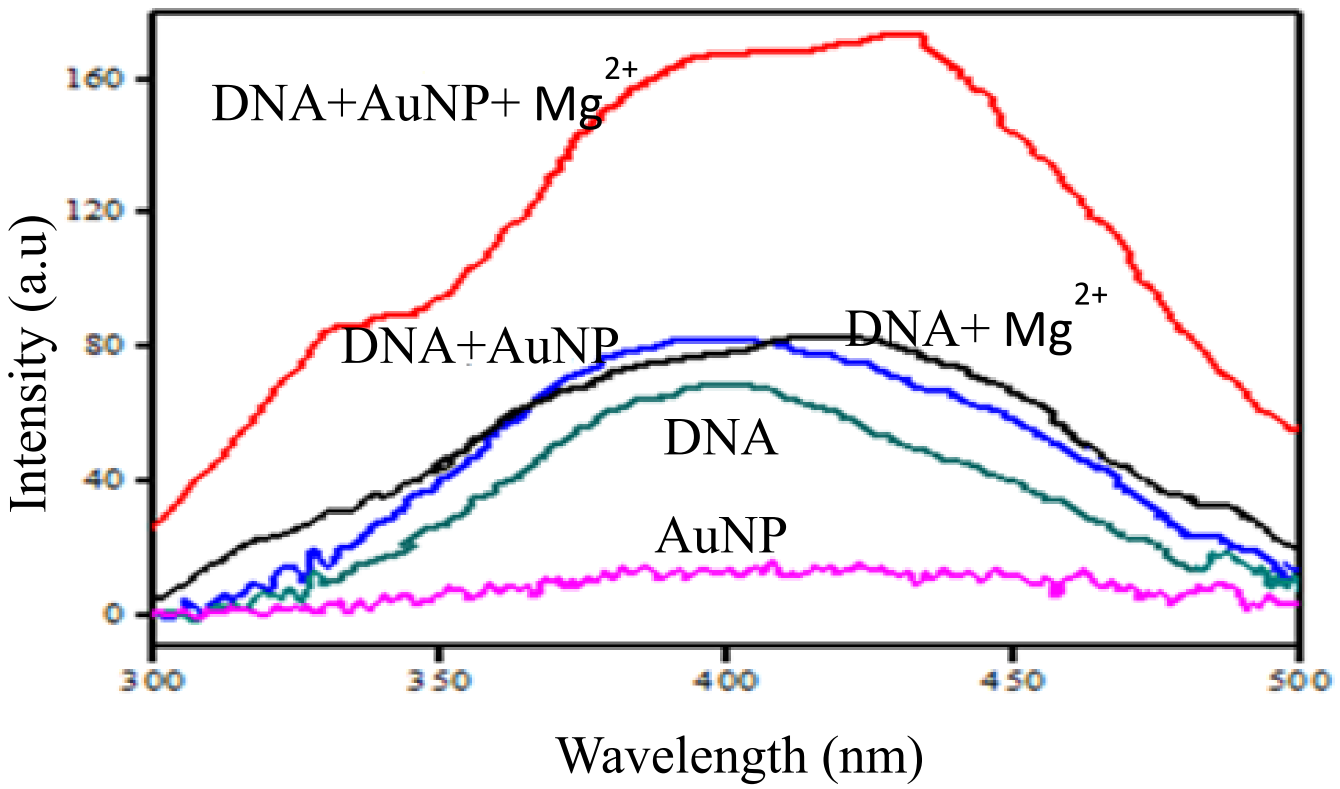

Figure S3: Fluorescence spectra of DNA-AuNPs with and without Mg^{2+} ions.

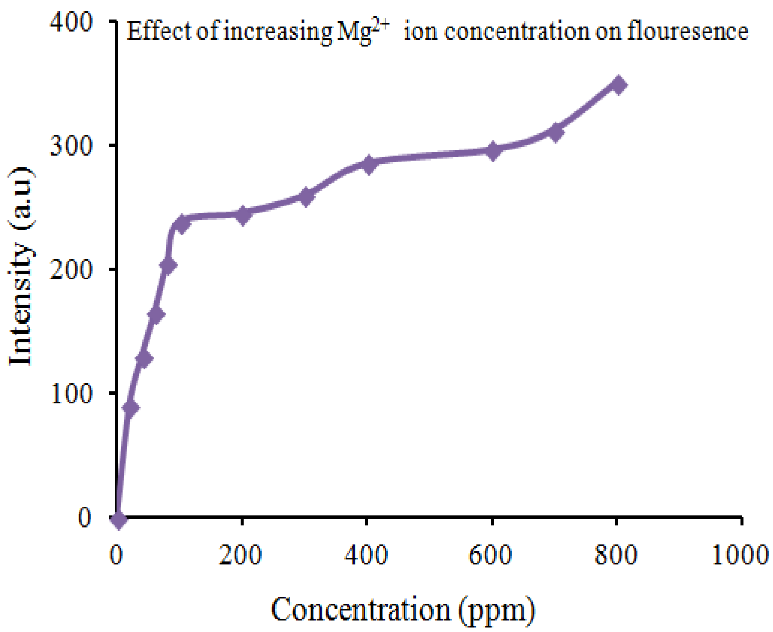

Figure S4: Graph showing change in fluorescence with increase in Mg^{2+} ion concentration.

Table S1: Showing shift in λ_{max} on adding DNA to AuNS.

S.No.	Sample	λ_{max} before addition of DNA (nm)	λ_{max} after addition of DNA (nm)	Shift in wavelength (nm)
1.	AuNS-1	524	538	14
2.	AuNS-2	541	544	3
3.	AuNS-3	611	627	15
4.	AuNS- 4	637	654	17
5.	AuNR	539, 697	552, 729	13, 28

Table S2**:** Variation in zeta potential, conductance and mobility of AuNP on addition of DNA.

S.No	Sample Name	Zeta Potential (mV)	Conductance (μS)	Mobility ($m^2V^1s\text{-}^1$)
1.	DNA	-16.84	183	- 1.28
2.	DNA + MPA	-7	20	1.78
3.	AuNS	+ 22.8	277	1.30
4.	AuNS + DNA	+16.67	270	- 6.17
5.	AuNR	+26.16	348	2.04
6.	AuNR + DNA	+10.40	305	0.81

Table S3**:** Determination of Mg^{2+} ion concentration.

S.No	Sample	Intensity	Mg^{2+} ions Conc. (ppm) obtained from standard graph (±5%)	Real concentration (ppm) of Mg^{2+} ions
1.	Gelusil	900 a.u	360	300
2.	Tap water	150 a.u	52	--
3.	Sample 1	180 a.u	62	50
4.	Sample 2	310 a.u	120	150
